# Case report: Treatment of advanced CSF1-receptor associated leukoencephalopathy with hematopoietic stem cell transplant

**DOI:** 10.3389/fneur.2023.1163107

**Published:** 2023-05-24

**Authors:** Caroline G. Bergner, Lisa Schäfer, Vladan Vucinic, Birthe Schetschorke, Julia Lier, Cordula Scherlach, Michael Rullmann, Osama Sabri, Joseph Classen, Uwe Platzbecker, Jörn-Sven Kühl, Henryk Barthel, Wolfgang Köhler, Georg-Nikolaus Franke

**Affiliations:** ^1^Department of Neurology, Leukodystrophy Clinic, University of Leipzig Medical Center, Leipzig, Germany; ^2^Medical Department, Hematology, Cellular Therapies and Hemostaseology, University of Leipzig Medical Center, Leipzig, Germany; ^3^Department of Radiology, University of Leipzig Medical Center, Leipzig, Germany; ^4^Department Pediatric Oncology and Hematology, University of Leipzig Medical Center, Leipzig, Germany; ^5^Department of Nuclear Medicine, University of Leipzig Medical Center, Leipzig, Germany

**Keywords:** ALSP, CSF1R-related leukoencephalopathy, white matter dementia, hematopoetic stem cell transplant, microglia

## Abstract

CSF1 receptor-related leukoencephalopathy is a rare genetic disorder presenting with severe, adult-onset white matter dementia as one of the leading symptoms. Within the central nervous system, the affected CSF1-receptor is expressed exclusively in microglia cells. Growing evidence implicates that replacing the defective microglia with healthy donor cells through hematopoietic stem cell transplant might halt disease progression. Early initiation of that treatment is crucial to limit persistent disability. However, which patients are suitable for this treatment is not clear, and imaging biomarkers that specifically depict lasting structural damage are lacking. In this study, we report on two patients with CSF1R-related leukoencephalopathy in whom allogenic hematopoietic stem cell transplant at advanced disease stages led to clinical stabilization. We compare their disease course with that of two patients admitted in the same timeframe to our hospital, considered too late for treatment, and place our cases in context with the respective literature. We propose that the rate of clinical progression might be a suitable stratification measure for treatment amenability in patients. Furthermore, for the first time we evaluate [18F] florbetaben, a PET tracer known to bind to intact myelin, as a novel MRI-adjunct tool to image white matter damage in CSF1R-related leukoencephalopathy. In conclusion, our data add evidence for allogenic hematopoietic stem cell transplant as a promising treatment in CSF1R-related leukoencephalopathy patients with slow to moderate disease progression.

## 1. Introduction

CSF1 receptor (CSF1R)-related leukoencephalopathy is a rare genetic disorder of cerebral white matter, leading to rapid neurological symptom progression including severe white matter dementia. The clinical picture and outstanding histopathologic features of CSF1R-related leukoencephalopathy were first described in the early 20th century as familial pigmentary orthochromatic leukodystrophy (1) and afterward as hereditary diffuse leukoencephalopathy with axonal spheroids (2). Later, the two diseases were discovered to share a common histopathologic and clinical phenotype designated as adult-onset leukoencephalopathy with axonal spheroids and pigmented glia [ALSP (3, 4)]. It has been shown that heterozygous variants in *CSF1R* gene account for a subset though not all ALSP cases as further genetic defects appear to convert on common histopathological features (5, 6).

The CSF1R transmits survival, chemotactic, and activation signals on myeloid cells (7). In the central nervous system (CNS), this receptor is expressed in microglia cells (8, 9). More than 100 mutations in *CSF1R* gene have been associated with the disease (10, 11), which were almost exclusively found within the receptor tyrosine kinase domain of the protein. Heterozygous variants in the *CSF1R* gene are assumed to alter microglia polarization and survival and thus CSF1R-associated leukoencephalopathy is a paradigmatic genetic microgliopathy (12, 13). The carrier of pathologic variants usually has a long asymptomatic disease course, and average symptom onset in patients occurs in the 4th decade. Clinically, the disease is characterized predominantly by cognitive decline and neuropsychiatric symptoms (executive dysfunction, changes in personality and behavior such as anxiety, depression, apathy, social disinhibition, irritability, and other frontal lobe symptoms) or motoric deficits such as parkinsonism and gait difficulty due to spasticity or severe gait apraxia. Epileptic seizures and stroke-like episodes are additionally found in some patients (14, 15).

While untreated, the disease is fatally progressive within few (~7) years, recently increasing numbers of cases worldwide have been reported in which allogenic hematopoietic stem cell transplant (allo-HSCT) has halted disease progression (16–19). These reports justify cautious optimism that functional deficits of microglial cells can be restored by replacing the patient’s hematopoietic system in its entirety and thus at least partially replacing the defective microglia through donor cells. Overall, experience with this novel treatment option is still very limited, and it is unclear up to which clinical stage patients might benefit from allo-HSCT. Sensitive detection of disease onset by imaging biomarkers is critical to enable early treatment and limit persistent deficits. On standard MRI sequences, abnormalities are often seen in asymptomatic carriers. Thus, there is a desire for alternative imaging tools providing readouts that are more specific in terms of the clinical phenotype. Molecular imaging of the myelin-binding tracer [18F] florbetaben might be promising in this regard. In this study, we report on two patients with CSF1R-associated leukoencephalopathy treated with allo-HSCT at advanced disease stages with progressed cognitive deficits adding to the growing body of evidence for the efficacy of allo-HSCT in this disease. We show for the first time that [18F] florbetaben myelin PET can depict affected areas, thus potentially representing an additive value over standard structural MRI and a suitable tool to monitor disease course/treatment effects on biological grounds.

## 2. Case histories

### 2.1. Case 1

A 47 year-old male presented at the center 4 years after first developing neurologic symptoms consisting of focal dysesthesia of the left arm and leg and visual deficits. After 2 years, he noticed gait disturbances and shrinking cognitive performance as well as slurred speech. When he presented at the center spastic gait disturbances, Parkinson-like movement of the lower limbs and right lower quadrantanopia were found, and cognitive decline was confirmed. Genetic evaluation revealed a heterozygous pathogenic variant in *CSF1R* gene (c.2342_2350del/p.Ala781_Asn783del). The variant was found in two of his four siblings as well as found post-mortem in his mother. His mother, grandmother, and two aunts had died at an early age from dementia. His older brother suffered from early onset (at 32 years) dementia in a progressed disease stage, while his younger, genetically affected brother was clinically and radiologically asymptomatic.

**Figure 1:**
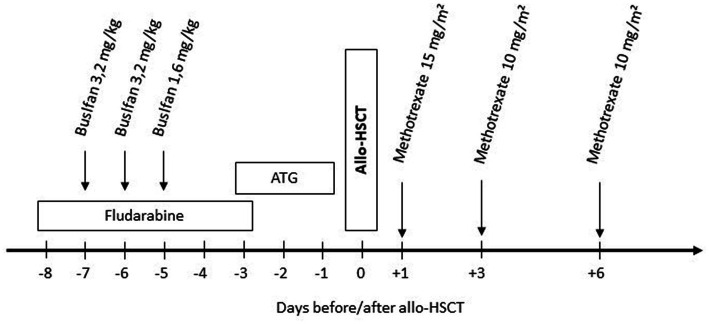
Conditioning regimen: fludarabine 30 mg/m^2^/day i.v. over 30 min (total 180 mg/m^2^). Busulfan 3,2/1,6 mg/kg body weight/d i.v. once daily over 3 h (total dose 8 mg/kg body weight) without busulfan kinetics/targeted AUC. Anti-thymocyte globulin (ATG, Grafalon^®^ Neovii, Switzerland) 30 mg/kg bodyweight/day i.v. over 6–12 h. Methotrexate 15 mg/kg body weight i.v. bolus on day +1 and 10 mg/kg body weight on day +3 and +6.

After reduced intensity conditioning with busulfan 8 mg i.v./kg bodyweight and fludarabine 180 mg/m^2^, allo-HSCT was performed using the peripheral blood stem cells from a 10/10 HLA-identical unrelated male donor. Immunosuppression consisted of *in vivo* T-cell depletion with ATG, cyclosporine A (CsA), and short-course methotrexate (Figure 1). CsA was tapered after 6 months and discontinued 9 months after allo-HSCT. Apart from mucositis II according to the WHO classification requiring transient intravenous pain medication and parenteral nutrition, no complications occurred. Full donor chimerism was noted on day +14 in the peripheral blood and was confirmed in the bone marrow on day +28 and remained stable in follow-ups (after 3 months, 1.25, and 2.5 years). No signs of acute or chronic GvHD were observed.

**Figure 2:**
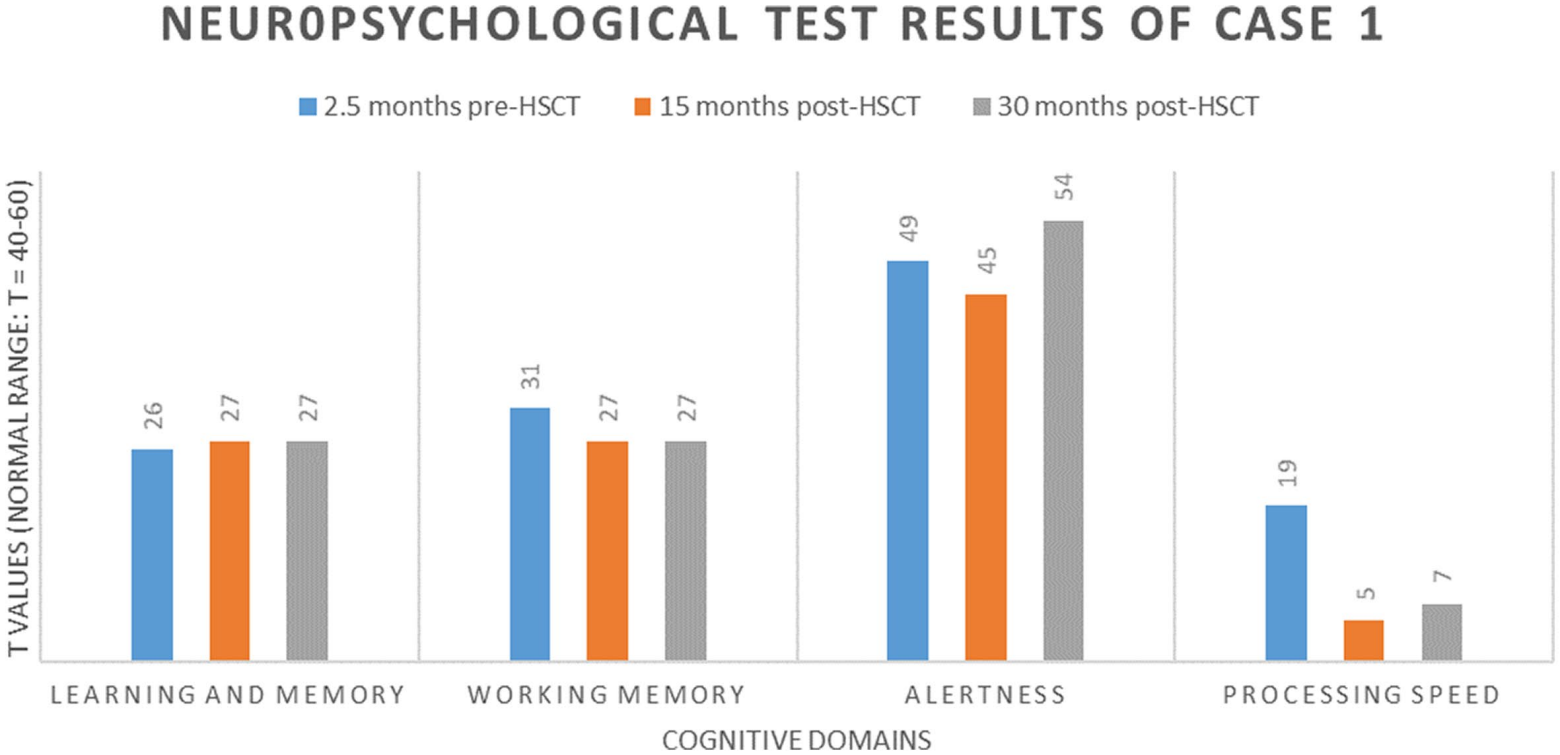
Neuropsychologic results of case 1.

In neuropsychological tests performed 2.5 months before allo-HSCT, the patient showed below-average performance (*T* ≤ 40) in non-verbal learning and memory (20), working memory (21), and processing speed (22) compared with age-, sex-, and education-based norm data and average reaction times in alertness tests [(23), Figure 2]. Testing 15 months after allo-HSCT showed a further cognitive decline in working memory, processing speed, and alertness, with performance on non-verbal learning and memory remaining stable after allo-HSCT. After 30 months of allo-HSCT, no further cognitive decline was observed.

Pre-transplant, the patient needed continuous bilateral assistance with maximum walking distance restricted to ~300 m. Shortly after therapy, gait disturbance further deteriorated, and the patient almost completely lost his walking ability during the first months after allo-HSCT. Starting from 4 to 6 months after the treatment, he slowly improved, reaching distances of 100 m with bilateral assistance 12 months after treatment that remained stable during the last follow-up at 30 months.

Pre-transplant MRI revealed confluent white matter lesions in the parietal lobe, central region, and along the pyramidal tracts combined with left-accentuated parietal atrophy. After 15 months of transplantation, a clear progression of the white matter lesions was shown, especially in the frontal areas associated with marked subcortical atrophy. No further progression was seen 2.5 years after transplantation neither in white matter lesions nor in atrophy.

**Figure 3:**
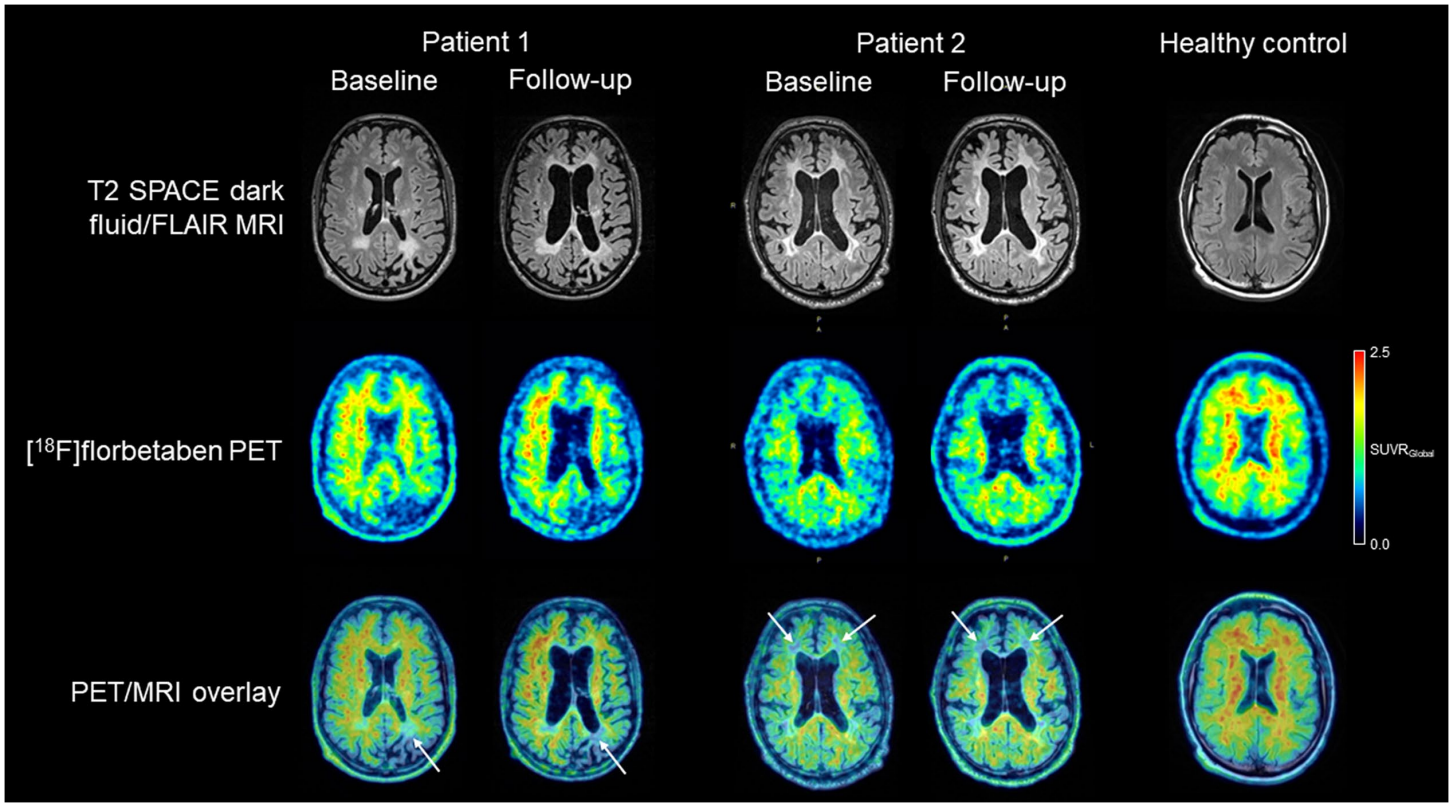
T2 SPACE dark fluid/FLAIR white matter integrity MR images, [18F] florbetaben myelin PET images, and overlaid PET/MR images of both adult-onset leukodystrophy with axonal spheroids and pigmented glia patients, together with (to illustrate respective imaging normality as a comparison) those of a healthy control subject. For the patients, baseline and follow-up images are shown. While in both patients with a favorable symptom outcome long-term after therapy, MRI showed progression of structural white matter integrity and subcortical atrophy, the Myelin PET images (white arrows) remained comparatively stable over time.

Florbetaben [18F] myelin PET imaging was carried out in this patient at the pre-transplant baseline as well as 2.5 years after the transplant. This was done after i.v. injection of ~300 MBq of the tracer on a Siemens Biograph mMR hybrid PET/3 T MRI system. PET images were acquired 90–110 min p.i. They were overlaid with the structural MRI data. Interestingly, it was observed that—different from the longitudinal MR images but keeping with the partial reversibility of symptom deterioration over this time-course— the white matter tracer binding reductions remained stable over time, without significant progress (Figure 3).

### 2.2. Case 2

A 42 year-old male patient had developed stuttering dysarthric speech in addition to gait and movement disturbance 2 years before the first presentation at the center. In the following year, cognitive deficits consisting of memory and concentration impairment were noticed, and gait imbalance also increased. When he first presented at the center 1 year later, 20 months prior to transplant, cognitive deficits were mild [montreal cognitive assessment (MoCA): 27/30 (24)]. On examination, gait apraxia and Parkinson-like movement disturbance were found. Genetic testing revealed a variant of unknown significance in *CSF1R* gene (c.2665A > C). His two brothers as well as his mother were negative for the variant, and his father had died early on of lung cancer without genetic testing.

Allo-HSCT was performed on his HLA-identical sibling after conditioning with busulfan 8 mg i.v./kg body weight and fludarabine 180 mg/m^2^ (Figure 1). Donor chimerism in the bone marrow was 100% on day 28 and 100% in the peripheral blood in the last follow-up 4 years after allo-HSCT. Persistent nausea and vomiting as well as acute kidney injury led to re-admission. Gastric and duodenal biopsies did not show signs of GvHD or infection. Symptoms resolved with symptomatic treatment and tapering of CsA, which was finally stopped 1.5 years after the allo-HSCT. The patient never developed acute or chronic GvHD.

At transplant, maximum walking distance without aid was limited to 500 m. Following allo-HSCT, patient’s relatives reported a rapid further deterioration, with walking restricted to a few steps and inability to walk stairs. In the cognitive screening of MoCA, he reached eight out of 30 total points 6 months after allo-HSCT. In particular, the patient showed severe deficits in executive functions (working memory, disinhibition, and apraxia), processing speed, attention, language (dysarthria), and short-term memory. Subsequent neuropsychological follow-up showed a relatively stable cognitive performance profile, with slight fluctuations only in the subtests of the MoCA that may be subject to daily fluctuations in attention [12/30 (15 months after allo-HSCT), 17/30 (30 months after allo-HSCT), 11/30 (42 months after allo-HSCT), and 15/30 (48 months after allo-HSCT)]. Furthermore, the patient regained pre-transplant walking ability 1 year after the transplant.

MRI imaging initially showed extensive frontoparietal white matter lesions. After 14 months of transplantation, a mild progression was seen into the temporal lobe combined with subcortical atrophy. In further follow-up MRIs, these lesions remained stable.

Florbetaben [18F] myelin PET imaging was carried out in this patient at THE at pretransplant baseline, 6 and 14 months after allo-HSCT. However, white matter tracer binding deficits remained relatively stable over this longer follow-up period (Figure 3).

### 2.3. Time course of cognitive decline in two untreated patients

Another two patients were admitted to the outpatient clinic in the same timeframe. A 43 year-old female presented 4 months after first neurologic symptoms consisting of memory loss were noticed by relatives. At the time of presentation, cognitive deficits were already fairly progressed [MoCA 16/30, Demtect 5/18 (25)], and the clinical condition was considered too advanced for treatment. The condition of this patient rapidly worsened reaching a vegetative state and finally leading to death only 2.5 years after symptom onset. A second patient presented 11 months after first symptoms were noticed, with severe cognitive deficits (MoCA 4/30) and was equally judged to be too advanced for treatment. This patient displayed a rapid decline in motor skills and cognition within few months. He lost communication skills and mobility and was not able to attend further follow-up visits at the center.

## 3. Discussion

CSF1R-related leukoencephalopathy is a rare genetic disorder, characterized by severe white matter degeneration leading to extensive white matter dementia, personality changes, and apraxia accompanied in most cases by motor deficits involving spastic gait, paresis sometimes mimicking stroke-like episodes, and parkinsonism. It has been inferred that CSF1R mutations might underlie 10%–25% of adult-onset leukodystrophies (11, 15). Thus, CSF1R-related leukoencephalopathy represents a major cause of genetic white matter dementia and must be considered in patients with rapid cognitive decline and motor impairment, specifically if family history of early-onset dementia or parkinsonism is present and white matter abnormalities are observed.

A total of 15 patients were reported worldwide in whom allo-HSCT was performed (16–19, 26–29). Therapy led to a favorable outcome in most of the individuals (14/15 survival >6months after treatment). All surviving patients showed stabilization of T2/FLAIR lesion load in long-term follow-ups. Cognition deteriorated in 5/15 patients after transplantation (no detailed information available in 3/15 patients, Table 1). We report on clinical features and outcome of two patients with CSR1R-releated leukoencephalopathy treated with allo-HSCT. Early therapy is assumed to be crucial to reach a favorable outcome as it holds true for further transplantable leukodystrophies, including X-linked adrenoleukodystrophy and metachromatic leukodystrophy (30–32) but has to be weighed against the mortality and potentially debilitating morbidity, especially graft-versus-host-disease [at least 10% even in young patients (33)]. Up to what stage transplantation can be performed safely in patients with CSF1R-associated leukoencephalopathy is an unanswered question. Recently, different clinical phenotypes were suggested to correlate with outcomes. Both patients treated in our institutions had initial motor symptoms that were proposed to show a better outcome compared to patients with primarily cognitive symptoms (16). In both our patients, neurological symptoms worsened intensively shortly after allo-HSCT, however, subsequently improved starting from nearly 6 months after transplantation, and clinical stabilization is now clearly stated by caregiving relatives though none of the patients is able to return to professional life or is independent in its activities of daily living. This transient deterioration is reported frequently in the literature and does not appear related to transplant-associated complications or transplant regimens (Table 1).

**Table 1:** Clinical characteristics thus far reported transplanted patients with CSF1R-leukoencephalopathy.

Patient	Reference	Initial symptoms	Time from disease onset to transplantation	Cognitive scores at transplantation	Cognitive scores at last follow up (timepoint after transplant)	Neuroradiologic outcome (timepoint after transplant of last assessment)	Conditioning//immunosuppression//donor	Complications//GvHD	Latest status
1	Case 1	Hemihypaesthesia, loss of vision	4 years	See Figure 2, SDMT: *T* = 19	SDMT: *T* = 7 (30 months)	First months after transplant with increase in T2/flair lesion load followed by long-term stabilization (30 months)	Bu 8 mg/kg BW, Flu 180 mg/m^2^//ATG, MTX, CsA//MSD (10/10)	Mucositis II°, acute kidney injury, diarrhea//none	Alive
2	Case 2	Stuttering speech, gait disturbances	5 years	20 months before transplant MoCA = 27/30	MoCA = 15/30 (48 months)	First months after transplant with increase in flair lesion load followed by long-term stabilization (42 months)	Bu 8 mg/kg BW, Flu 180 mg/m^2^//ATG, MTX, CsA//MUD (10/10)	Fall with fractures//none	Alive
3	(26)	Impulsivity, cognitive deficits	No detailed information available	N.a.	N.a.	N.a.	N.a.// N.a.//N.a.	N.a.//N.a.	N.a.
4	(17)	Slowness of thinking	9 years	MoCA = 21/30	MoCA aproximately 23/30 (30 months)	First months after transplant with slight increase in flair lesion load, followed by long-term stabilization, resolution of DWI abnormalities (28 months)	Cy/Flu/TBI//PT-Cy, Tac, MMF//haplo-identical donor	Pneumonia//none	Alive
5	(17)	Depression, memory deficits	12 years	MMSE = 19/30; (approximately, not completely clear)	MMSE = approximately 18, (27 months)	First months after transplant with partly reversible increase in T2 lesion load, followed by long-term stabilization, resolution of DWI abnormality (30 months)	MAC//ATG, MTX, Tac//MUD (12/12)	UTI, seizures//none	Alive
6	(19)	Memory loss and disinhibition	3 years	STMS = 27/38	STMS = 11/38 (27 months)	First months after transplant with increase in lesion load and atrophy followed by long-term stabilization (27 months)	N.a.//N.a.//MUD	N.a.//GvHD II° requiring steroids	Alive
7	(19)	Cognitive decline, spasticity, tremor	2 years	MMSE = 20/30	MMSE = 10/30 (20 months)	First months after transplant with increase in lesion load and atrophy followed by long-term stabilization (20 months)	N.a.//N.a.//MUD	N.a.//none	Alive
8	(19)	Gait disturbances	2 years	STMS = 31/38	STMS = 34/38 (3 months, no long-term results available)	Post-transplant increase in lesion load and atrophy (9 months) long-term examination n.a.	N.a.//N.a.//MSD	N.a.//none	Alive
9	(19)	Ataxia, impaired verbal fluency	2 years	MMSE = 24/30	MMSE = 29/30 (15 months)	Stable T2/flair lesion load, Slight increase in atrophy on long-term follow up (15 months)	N.a.//N.a.//N.a.	None//N.a.	Alive
10	(19)	Memory deficits	3 years	MMSE = 29/30	MMSE = 26/30 (7 months)	Stable T2/flair lesion load, slight increase in atrophy on long-term follow-up (11 months)	N.a.//N.a.//N.a.	Left-toe cellulitis, BK virus cystitis, and small left-sided subdural hematoma//N.a.	Alive
11	(19)	Personality changes	N.a.	STMS = 27/38	N.a.	N.a.	N.a.//N.a.//N.a.	Acute kidney injury, cardiac arrest	Died on day +88
12	(19)	Behavioral changes	N.a.	MMSE = 24/30	MMSE = 20/30 (6 months)	Stable (6 months), long-term data n.a.	N.a.//N.a.//N.a.	N.a.//GvHD II°, requiring steroids	Alive at 6 months
13	(18)	Motor symptoms	N.a.	MoCA = 28/30	MoCA = 26/30 (30 months)	First months after transplant with partly reversible increase in T2 lesion load, followed by long-term stabilization, resolution of DWI abnormality (30 months)	MAC using Bu/Flu//PT-Cy, ATG, CsA and MMF //5/10 haplo-identical sister	Hemorrhagic cystitis, pyelonephritis//none	Alive
14	(29)	Gait disturbances, dysarthria	3 years	Mild cognitive impairments, no quantification	N.a.	Long-term stabilization of T2 lesion load (22 months)	Bu 130 mg/m^2^/Flu 150 mg/m^2^//N.a.//MUD	UTI, mood disorder, suicide attempt 22 months after HSCT//none	Alive
15	(27)	–	Presymptomatic	Presymptomatic treatment	Stable	Stable	N.a.//PT-Cy, sirolimus, MMF//UD (9/10)	Transient inflammation of the left temporal lobe, brainstem, medulla, and meninges//none	Alive

ATG, anti thymocyte globulin; Bu, busulfan; BW, body weight; CsA, cyclosporine A; Flu, fludarabine; HSCT, hematopoietic stem cell transplantation; MAC, myeloablative conditioning; MMF, mycophenolat mofetil; MMSE, mini mental status examination; MoCA, montreal cognitive assessment; MSD, matched sibling donor; MTX, methotrexate; MUD, matched unrelated donor; PT-Cy, post-transplantation cyclophosphamide; SDMT, symbol digit modalities Test; STMS, short test of mental status; Tac, tacrolimus; UD, unrelated donor; UTI, urinary tract infection; n.a, not available.

CSF1R-related leukoencephalopathy is a rapidly progressing disease, with on average 6–8 years between the first symptoms and death, but disease duration can be highly variable [ranging from 1 to 29 years (11, 34)]. The time from disease onset to allo-HSCT was 4 years in patient 1 and 5 years in patient 2, and still at the time of transplantation, clinical deficits were moderate. A comparable progression rate for cognitive symptoms from disease onset to transplant time was found for all other transplanted patients reported so far in the literature using comparable neuropsychological screenings like MoCA or the mini-mental status test (Table 1). In contrast, fairly rapid disease courses were found in two other patients with initial cognitive symptoms admitted in the same time frame at our institution, judged too late for treatment. Given the literature (34) and our patients, we would therefore suggest that the pre-transplant rate of progression of cognitive decline rather than a patient’s momentary levels of clinical disability are relevant outcome predictors that should guide allo-HSCT treatment decisions for CSF1R-related leukoencephalopathy.

Identification of asymptomatic carriers, higher awareness of patients and relatives, as well as suitable screening programs might improve early diagnosis, leading to presymptomatic identification of patients at risk. There is hope, that imaging or liquid biomarkers, such as neurofilament light chain (35) will allow a better presymptomatic diagnosis of disease onset that could open up treatment windows also for rapidly progressing patients. Patchy white matter alterations are typical features of early disease, progressing to confluent lesions, often with frontal predominance, persistent diffusion restriction, and thinning of the corpus callosum in the further disease course (10, 15, 36, 37). Standard MRI sequences such as T1-, T2-or fluid-attenuated inversion recovery have been shown to correlate with disease progression (38). Importantly, however, the corresponding tissue correlate is not well defined, and alterations may be present in a subset of patients at asymptomatic stages of the disease where they can remain stable over years. In the study, imaging approaches more specifically picturing cells and tissues of interest are needed. Molecular imaging using the myelin-binding PET tracer [18F] florbetaben might be able to fill this gap. This tracer was developed to image beta-amyloid aggregates in the neocortex of patients with Alzheimer’s disease (39). In addition to this feature, the tracer binds, in white matter regions, to myelin (40). In fact, we demonstrated in our patients that [18F] florbetaben PET is able to depict extended areas of white matter degeneration in CSF1R-related leukoencephalopathy. Interestingly, long-term follow-up imaging resulted, in contrast to the MR imaging, in rather stable findings. Thus, it appears that the molecular imaging technique of [18F] florbetaben PET provides additional information and given the transient nature of clinical deterioration might even be a better surrogate readout as compared to MRI in terms of the clinical phenotype. Future studies are needed to systematically extend our findings correlating longitudinal PET results with those obtained by MRI, clinical disease stages, and eventually post-mortem histopathology.

In summary, our cases add to the growing body of evidence on allo-HSCT representing a suitable treatment in CSF1R-related leukoencephalopathy, capable of halting brain damage and symptom progression, including severe white matter dementia and motor impairment. However, treatment of rapidly progressing patients remains challenging even after the incorporation of novel biomarkers in clinical follow-ups. Thus, there is an unanswered need for the development of alternative treatments, less risky than allo-HSCT that might be applied at pre-symptomatic stages or even in asymptomatic carriers, and the feasibility of preventive allo-HSCT has to be further explored. Educating physicians in neurocognitive clinics as well as primary treating neurologists about typical disease features of CSF1R-related leukoencephalopathy, novel therapeutic options, and time sensitivity of diagnosis is of utmost importance.

## Data availability statement

The datasets presented in this article are not readily available because of ethical and privacy restrictions. Requests to access the datasets should be directed to the corresponding authors.

## Ethics statement

Ethical review and approval was not required for the study on human participants in accordance with the local legislation and institutional requirements. The patients/participants provided their written informed consent to participate in this study.

## Author contributions

CB, LS, WK, JL, and JC oversaw neurologic clinical care of the reported patients while G-NF, VV, BS, and UP conducted hematopoietic stem cell transplant and hematologic follow-ups. CS oversaw and reported MRI data. MR, HB, and OS conducted and analyzed PET data. CB, G-NF, LS, HB, and WK drafted and discussed the manuscript. All authors contributed to the article and approved the submitted version.

## Conflict of interest

The authors declare that the research was conducted in the absence of any commercial or financial relationships that could be construed as a potential conflict of interest.

## Publisher’s note

All claims expressed in this article are solely those of the authors and do not necessarily represent those of their affiliated organizations, or those of the publisher, the editors and the reviewers. Any product that may be evaluated in this article, or claim that may be made by its manufacturer, is not guaranteed or endorsed by the publisher.
